# Finite Element Modelling of Pulsatile Blood Flow in Idealized Model of Human Aortic Arch: Study of Hypotension and Hypertension

**DOI:** 10.1155/2012/861837

**Published:** 2012-02-13

**Authors:** Paritosh Vasava, Payman Jalali, Mahsa Dabagh, Pertti J. Kolari

**Affiliations:** ^1^Faculty of Technology, Lappeenranta University of Technology, P.O. Box 20, 53851 Lappeenranta, Finland; ^2^South Karelian Institute, Lappeenranta University of Technology, P.O. Box 20, 53851 Lappeenranta, Finland

## Abstract

A three-dimensional computer model of human aortic arch with three branches is reproduced to study the pulsatile blood flow with Finite Element Method. In specific, the focus is on variation of wall shear stress, which plays an important role in the localization and development of atherosclerotic plaques. Pulsatile pressure pulse is used as boundary condition to avoid flow entry development, and the aorta walls are considered rigid. The aorta model along with boundary conditions is altered to study the effect of hypotension and hypertension. The results illustrated low and fluctuating shear stress at outer and inner wall of aortic arch, proximal wall of branches, and entry region. Despite the simplification of aorta model, rigid walls and other assumptions results displayed that hypertension causes lowered local wall shear stresses. It is the sign of an increased risk of atherosclerosis. The assessment of hemodynamics shows that under the flow regimes of hypotension and hypertension, the risk of atherosclerosis localization in human aorta may increase.

## 1. Introduction

Atherosclerosis is the disease of large arteries (carotid, aorta, and other proximal arteries) and tends to localize in regions of curvature and branching in arteries. Caro et al. [1, 2] and DeBakey et al. [3] categorized aortic arch, major branches of aortic arch, and abdominal aorta as susceptible sites for creation and development of atherosclerosis. The complex anatomies of arteries are often associated with abnormal flow dynamics and stress distributions. Ku et al. [4], Nerem [5], Tarbell [6], and Zarins et al. [7] have reported that atherosclerosis is more prone to occur in regions of low shear stress and oscillating shear stress increases the risk of atherosclerosis localization. Fry [8] demonstrated that under acute elevation of shear stress the endothelial layer of arterial wall may damage and increases its permeability for lipids. These studies prove that there is a positive correlation between low and fluctuating wall shear stress and risk of development of atherosclerosis.

The human aorta has complex anatomy with curvature, branching, and distal tapering. Caro [2] and Fry [8] have shown that the human aortic arch is vulnerable to localization of atherosclerosis due to the complex anatomy of aorta. In his work, Utepov [10] has demonstrated the tapering of arteries as one of the risk factors for manifestation of atherosclerosis. Kilner et al. [11] detected, in vivo, the complicated helical and retrograde flow caused by the aortic arch and pulsatile nature of the inflow of blood from left ventricle of heart. Thus the complex arterial flow mechanics in aorta may promote the early development atherosclerotic plaque. The blood flow in thoracic aorta models has been also studied numerically [12–19]. However, some of studies neglected the three major of branches of aortic arch. Towfiq [9] and Dabagh et al. [20] have shown that the aorta size is subjected to change with blood pressure. But no attention has been made on studying the corresponding influence on the blood flow features. Moreover, the effect of blood pressure on changing the aorta geometry has also been ignored.

In the present study, we have reconstructed a three-dimensional (3D) aorta model adopted from the literature. The aorta model includes three major branches (brachiocephalic artery, left carotid artery and left subclavian artery) in the aortic arch. The pulsatile blood flow through three 3D model of aortic arch was simulated with incompressible Navier-Stokes equations. The governing equations along with boundary conditions are solved with Finite element method- (FEM-) based code Comsol Multiphysics V3.4. The idealized aorta model along with inlet pressure profile was further modified to simulate aorta under hypotension and hypertension conditions. Though the transient variation of blood pressure within a cardiac cycle causes instantaneous deformation of the aorta wall, which in turn may affect the blood flow features (two-way coupling between wall deformation and blood flow), such effects are ignored in this study. Because of the critical role played by the shear stress in arterial wall diseases, the wall shear stress (WSS) is studied extensively within branches and through the arch of aorta. Moreover, the velocity profiles across various cross sections of aortic arch and branches are investigated. The first objective of present study is to find out how the aortic geometry is connected with the blood flow development and the shear stress distribution in the aorta wall. The second objective is to realize how blood pressure and its associated wall deformation affect the distribution of WSS, the flow profiles, and the volumetric outflow across branches. Therefore, this study seeks the role of aortic arch geometry (with branches) and the mean blood pressure on the resulting transient blood flow only when the wall of aorta is rigid. However, one should note that the corresponding results might be different than what we observe in reality due to the flexibility of real wall and the complexity of the real aorta geometry.

## 2. Materials and Methods

**Figure 1:**
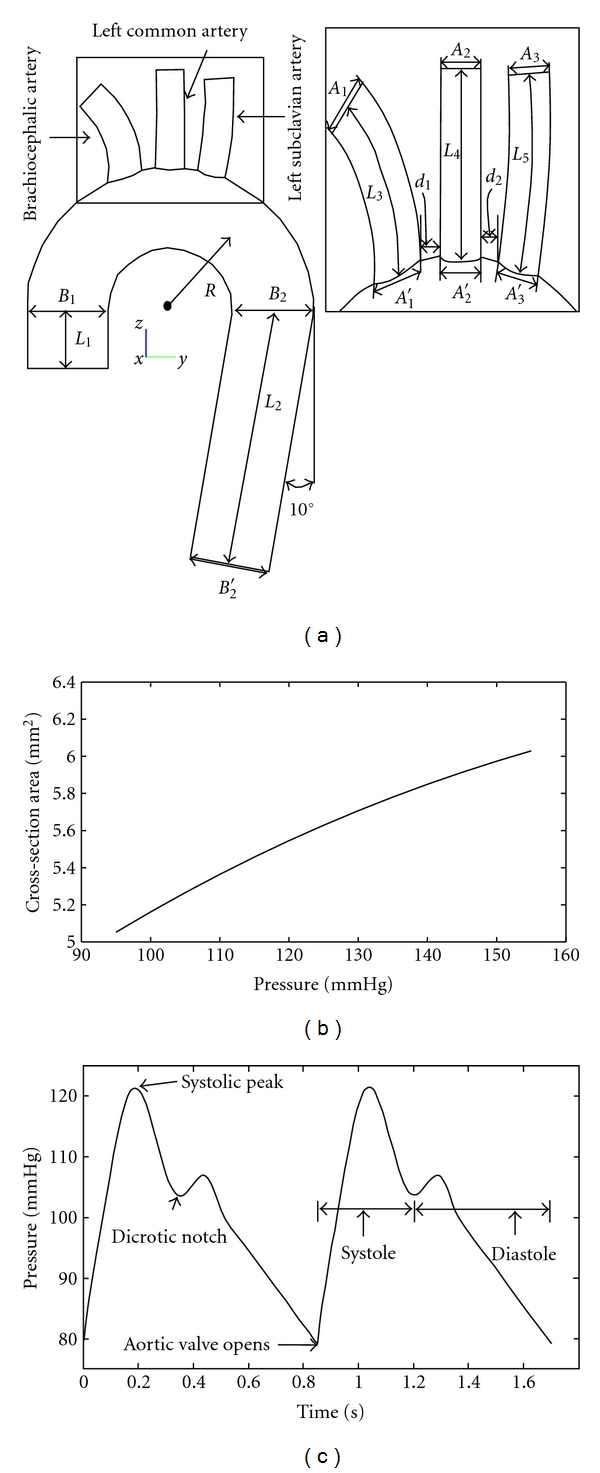
(a) Schematic diagram of 3D aortic arch model (Inset: a zoomed region around branches with definitions of lengths, diameters, and distances also common with the two other geometries), (b) the plot of luminal cross-sectional area of ascending aorta versus pressure estimated from Towfiq et al. [9]. (c) Pressure pulse at the inlet of ascending aorta under normal pressure condition.

The idealized model of human aorta was reconstructed based on aorta model used by Shahcheranhi et al. [18]. The model is constructed in six parts, namely, ascending aorta, aortic arch, descending aorta, and three branches (brachiocephalic artery, left common carotid artery, and left subclavian artery). The simplification of the geometry is basically associated with neglecting the undetermined curvature of the abdominal part of aorta and torsion of ascending aorta, as the original aorta models were reconstructed from the computed tomographic (CT) images. Moreover, there may be minor differences in original aorta models and aorta models used in this study due to different design module used for reconstruction purpose.  Figure 1(a) illustrates the schematic diagram of aorta with the details of geometric measures given in Table 1. The aorta model was modified for two working pressures inside the aorta mimicking the hypotension (65–105 mmHg) and hypertension (100–140 mmHg) conditions. The variation of the inlet cross-sectional area versus pressure is demonstrated in Figure 1(b), which has been used in the construction of the geometry under the effect of hypotension and hypertension conditions.

The blood is assumed to be a Newtonian fluid. The assumption of Newtonian fluid for blood with a constant viscosity is feasible in large arteries. Although some works such as Khanfar et al. [21] showed that the non-Newtonian assumption of blood affects the blood flow in aorta aneurysms, their simulations did not display significant differences in shear stress calculated from Newtonian and non-Newtonian simulations. The driving force for the blood flow in an artery is the pressure gradient along the artery. Thus the pulsatile pressure-inlet boundary condition (with zero viscous stress) was used at inlet and outlets. The outflow from left ventricle of heart is not always uniform as flow disturbance is induced by aortic valve. Although an exact effect of tricuspid aortic valve cannot be exactly mimicked, with the deployment of pressure-inlet boundary condition we can avoid uniform inflow at inlet of aorta which has been typically used in several works. Thus the pressure-inlet boundary condition in the present study will be more realistic. In the present study, a pulsatile pressure is deployed as inlet and outlet boundary conditions. An eighth degree polynomial was used to reproduce the inlet pressure pulse in mmHg from the data given by Conlon et al. [22] as

(1) $$f\left(t\right)=\{\begin{array}{cc} \overset{9}{\underset{i=1}{\sum }}C_{i}{\left(t-0.85n\right)}^{i}+79.20 & \\ \;\;\text{if}\;t\in \left[0.85n,\;0.85\left(n+1\right)-0.34\right], & \\ -61.50\left(t-0.85n\right)+131.47 & \\ \;\;\text{if}\;t\in \left[0.85\left(n+1\right)-0.34,\;0.85\left(n+1\right)\right], & \end{array}$$

where *n* is the cardiac cycle number varying from 1 to 4. The polynomial coefficients *C*_*i*_ are introduced in Table 2. The period of systole and diastole in the pressure pulse took 0.35 s and 0.5 s, respectively. The pressure pulse at the inlet of ascending aorta is shown under normal pressure condition in Figure 1(c). For boundary conditions at the outlets, the pressure pulse is multiplied by a certain coefficient that represents pressure drop between inlet and respective outlet. The coefficients for respective branch outlets are obtained by a separate series of steady-state simulations with flat velocity at inlet and target mass flow at outlet. Li [23] has suggested that in large arteries it is reasonable to assume that the blood close to artery moves with same speed as that of wall. Thus over the walls of aorta, no-slip boundary condition is applied. Under transient condition and the assumption of incompressible flow with Newtonian rheology, the flow of blood in the aorta is governed by the continuity and Navier-Stokes equations

(2) ∇·u=0,

(3) $$\rho \frac{\partial u}{\partial t}\;+\rho \left(u.\nabla \right)u\;\;=-\nabla p+\mu \;\nabla .\left(\nabla u+{\left(\nabla u\right)}^{T}\right),$$

where **u **denotes fluid velocity vector and *p* represents hydrostatic pressure. Also, *ρ* is the density of blood taken as 1060 kg/m^3^ and *μ* is the dynamic viscosity of blood that is a constant as 0.005 Pa.s. 

To ensure periodic nature of the flow, the simulations were performed for four cardiac cycles (pressure pulse) where each cycle is 0.85 s. The results from fourth cardiac cycles are discussed in the results section. The governing equations were discretized with Backwards Difference Method, which is known to be a very stable method for discretization. The set of governing (2)-(3) along with boundary conditions were solved by means of the FEM method provided in COMSOL Multiphysics, v. 3.4. The mesh grid for each aorta case was refined subsequently to obtain mesh-independent-results. The computational mesh utilized in primary simulations consisted of nearly 35000 to 42000 tetrahedral elements. The mesh grid was refined till the resulting distributions of the flow and WSS were qualitatively identical: spatially and temporally. The final mesh grid consisted of nearly 140000 grid elements. To further increase accuracy and reliability of the solutions, the local variations of flow variable within the grid elements were predicted by quadratic piecewise functions. Memory friendly iterative solvers GMRES and FGMRES were used for solving the discretized governing equations. The residual for solution was kept at 1 × 10^−4^ and the simulations progressed with a time step of 0.001 s. A steady-state simulation with 80 mmHg pressure was performed with direct solver UMFPACK and was used as an initial guess for the transient simulations. The transient simulations were performed with personal computer with 3 GHz Core 2 Duo processor with 3 GB of RAM.

The aorta model used in the current study was adopted from the literature and thus lacked some features of realistic aorta, for example, curvature in descending aorta. The study was focused on flow dynamics in aortic arch and three major branches, thus the branches of thoracic aorta were neglected. In real aorta, the branch entry regions have blunt corners; however, this feature of aorta model was not included due to lack of exact measurements. This feature of aorta model was also found missing in the original works [13, 18]. Due to limited memory available for computation, the fluid-structure interaction could not be included in the current set of simulation. The reported study is mainly focused on understanding the effects of hypotension and hypertension on the flow and WSS distributions. The reported study is mainly focused on understanding the effects of hypotension and hypertension on the flow and WSS distributions. Although it is indicative that assumption of rigid wall in blood flow simulation may underestimate temporal and spatial flow and wall motion, the aorta walls were assumed to be rigid for flow simulations under all three pressure regimes. Long-term hypertension can cause thickening of arterial wall and loss of elasticity of arterial wall [24]. Thus, aorta walls can be assumed rigid under hypertensive flow regime. The assumption of rigid artery wall in other two cases is acceptable as earlier works [21, 25–27] have demonstrated that the arterial compliance does not influence general characteristics of flow and WSS significantly. Moayeri and Zendehbudi [25] compared hemodynamic characteristics of blood flow through arterial stenosis with and without distensible walls numerically. The comparison revealed no difference between WSS distributions for the rigid and deformable walls during systole and diastole. Zhao et al. [27] studied the influence of wall distensibility on WSS numerically and did not found any significant difference between the WSS in rigid and compliant models. It is of notice that Zhao used an angiogram-based model of carotid bifurcation for their experiments. The localization of atherosclerosis is often localized in regions of low and oscillating WSS, flow stagnation, or recirculation. The elongation of separated flow region and local migration of recirculation or stagnant flow region may influence the localization of atherosclerosis only locally.

## 3. Results and Discussion

The axial velocity distributions and peripheral WSS at peak systolic time instance *t* = 0.18 s are compared. 

### 3.1. The Distributions of Pressure, Flow Field, and WSS for Normal Pressure Case

**Figure 2:**
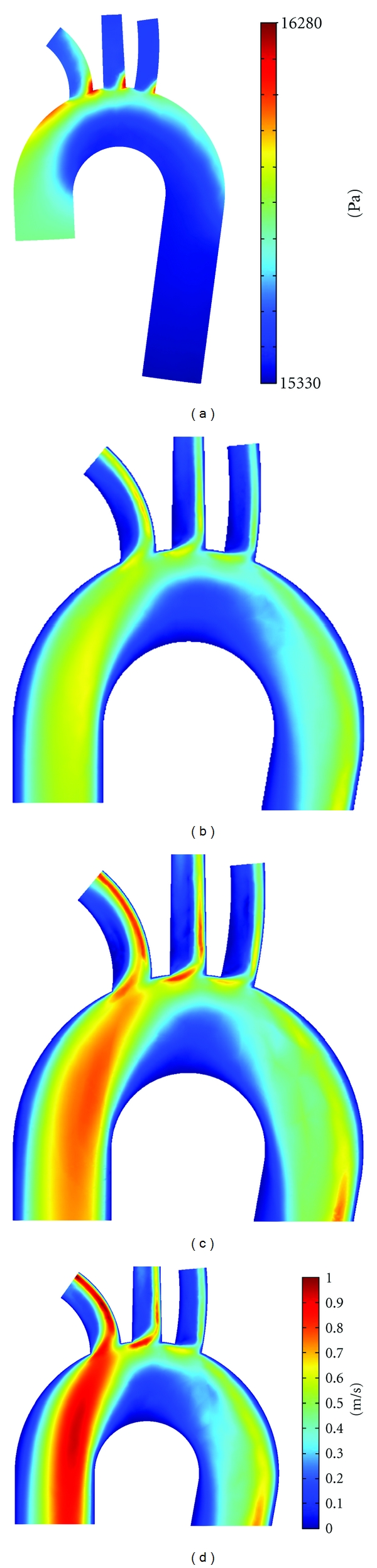
(a) Distribution of pressure for normal pressure regime, velocity distributions across the symmetry plane of the aorta model under (b) hypotension, (c) normal pressure, and (d) hypertension at peak systolic at time instance *t* = 0.18 s.

The pressure iso-contours and distribution of velocity across the coronal plane captured at time *t* = 0.18 s are shown in Figures 2(a) and 2(c), respectively. In aortic, arch high pressure is distributed on the upper aortic arch while low pressure is distributed at the inner curvature of the arch. The most prominent feature of pressure distribution is the maximum pressure distributed at the branch entry region of three branches. The maximum pressure is distributed on the distal side of three branch entry regions, while relatively lower pressure is distributed on the proximal side of the branching regions.

The axial flow in ascending aorta is marginally skewed towards the outer wall of the aorta. The marginal skewness persists close to the proximal aortic arch as well. In the aortic arch, the flow close to the inner aortic arch slows down while the flow in the upper aortic arch accelerates. At the flow stagnation region in the aortic arch, the velocity of the flow is less than 0.01 m/s, which may account for longer particle residence time of blood cells and thus the localization of atherosclerosis. The pressure gradient across the branch entry region causes the maximum velocity of magnitude about 0.72 m/s on the proximal side of the branch entry region of brachiocephalic artery (Figure 2(c)). The flow separation and secondary flow motion in aortic arch affects the flow in distal aortic arch as well. In region after the left subclavian artery (the third branch), the flow profiles display more tendency to the distal wall. The flow in distal aortic arch and descending aorta is extremely skewed towards the inner wall. 

**Figure 3:**
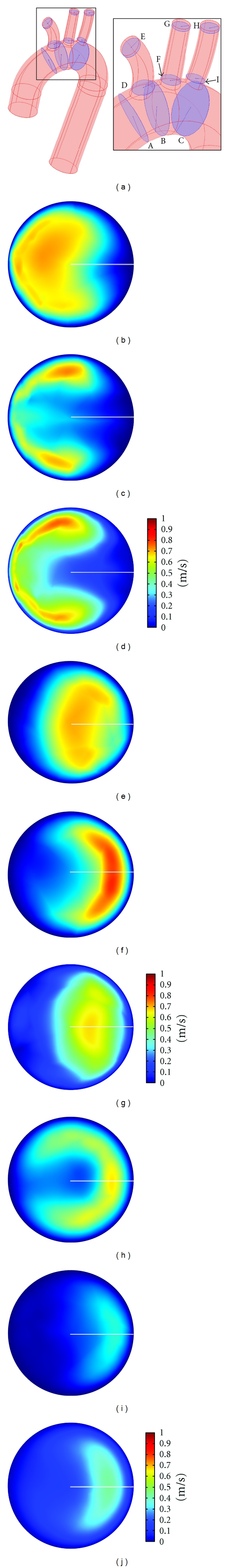
(a) Axial cross-sections within the aortic arch and branches; axial velocity profiles across axial cross-section, (b) A, (c) B, (d) C, (e) D, (f) E, (g) F, (h) G, (i) H, and (j) I in the aortic arch model captured at peak systolic time *t* = 0.18 s.

To obtain details of the secondary flow motion in the aortic arch and branches, velocity profiles across axial cross-sections of the aortic arch and branches (as shown in Figure 3(a)) were captured. The axial flow profiles for normal pressure case are demonstrated in Figures 3(b)–3(j). At section-A (Figure 3(b)), the flow is skewed towards the inner wall of the aortic arch. The aortic arch curvature induces secondary flow motion, which were captured at sections-B and C. The flow near outer wall of the arch is directed towards the inner wall of the arch causing *ε*- and C-shaped velocity distributions at sections B and C, respectively. At sections B and C, the flow is concentrated substantially around the outer wall of the arch. It is also of notice that the flow velocity retards as it moves from top towards the bottom of the axial section. 

The axial flow distributions at sections D, F, and H suggest significant disturbance at the branch entry regions. This is demonstrated in Figures 3(e), 3(g), and 3(j). This is caused by the bifurcating flow coupled with pressure gradient across branch entry region. At sections D, F, and H, maximum velocity is distributed towards the distal side of the branch. The pressure gradient across the axial cross-section of braches causes flow separation and secondary flow patterns in branches as well. In Figure 2(c), it can be noticed that the flow in branches is skewed towards the proximal walls. The skewness of the flow causes flow stagnations close to the proximal walls of respective branches. It is also noticeable that at the axial sections at the branch exit region (sections E, G, and I), the flow remains localized towards the distal wall. The pressure gradient between proximal and distal wall is highest in brachiocephalic artery, moderate in left carotid artery, and lowest in left subclavian artery. The effect of weakening pressure gradient is also evident in axial flow profiles in sections E, G, and I. It can be observed that the magnitude of velocity is highest in brachiocephalic artery (section E), moderate in moderate in left carotid artery (section G), and lowest in left subclavian artery (section I). During reconstruction of aorta model, the effect of tapering is incorporated in the carotid artery. The tapering effect causes overlapping of pathlines close to outlet of the carotid artery. As a result of this, the axial flow distribution in section G is almost *O*-shaped, while in sections E and I the axial flow distributions are *C*-shaped. 

**Figure 4:**
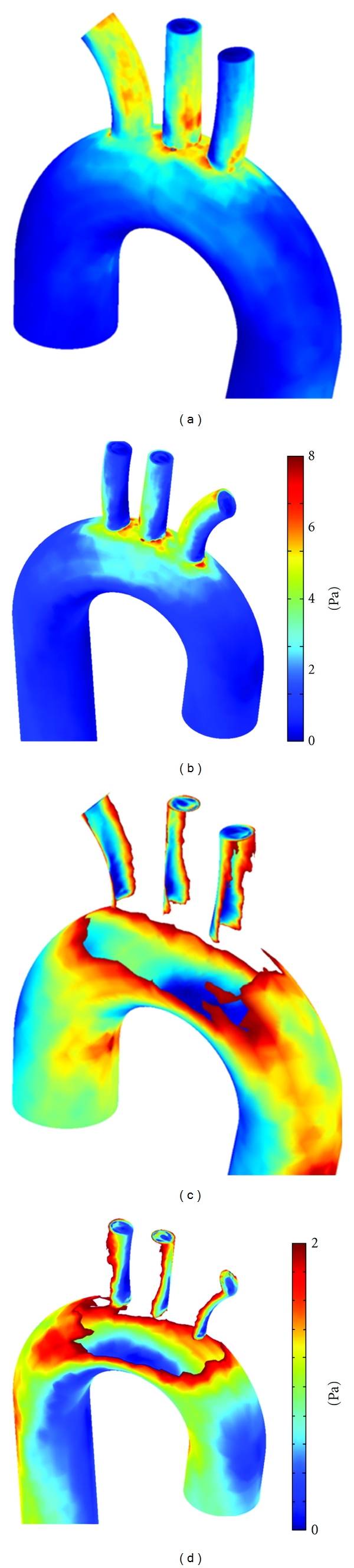
Anterior (a, c) and posterior (b, d) views of aorta model showing WSS distribution over the aortic arch and branches at time *t* = 0.18 s.

The distribution of velocity in vicinity of the walls is crucial for the calculating WSS, as low and fluctuating WSS are known to be the key parameters of the development of atherosclerotic plaques. Higher WSS is also known to be closely related to aorta dissection. Ku [24] has reported that skewness in axial profiles can cause alteration and oscillation of local WSS, which can cause localization of atherosclerosis. The distributions of WSS at peak systole for aorta model under normal pressure regime are presented in Figure 4. The distributions are shown in two view angles with full and low range of WSS. The anterior and posterior views in Figure 4(a) and Figure 4(b) suggest lower WSS distributed on the proximal walls of branches and the higher WSS distributed on distal walls of branches. On lowering down the scale we also realized extremely low WSS distributed at the outer wall or ascending aorta and inner wall of the aortic arch (Figure 4(c) and Figure 4(d)). The demonstrated distribution of flow and WSS were in qualitative agreement with the earlier numerical [13, 18, 28] and experimental [5] works.

**Figure 5:**
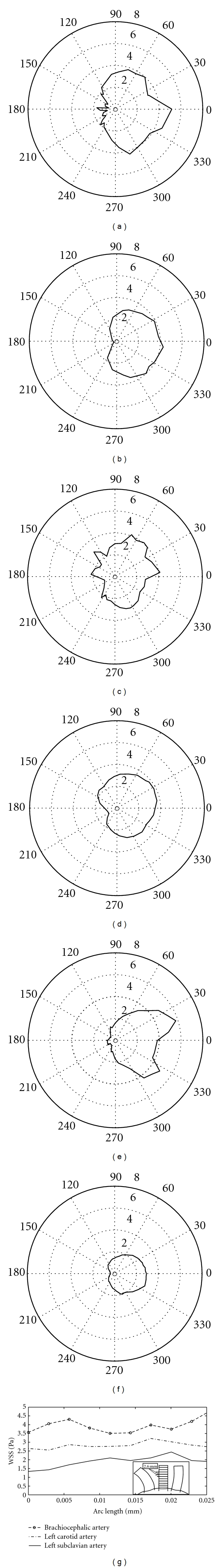
Polar plot of peripheral WSS distribution over the axial section (a) D, (b) E, (c) F, (d) G, (e) H, (f) I, and (g) WSS averaged over length of branch arteries at time *t* = 0.18 s.

To further analyze the WSS distributions quantitatively, circumferential WSS captured at axial cross-sections D–I is plotted as polar plots in Figure 5. In sections D, F, and I of branches, the low values of WSS are distributed in the angular domain from 90° to 270° counterclockwise, while the higher values of WSS are distributed on the opposite domain, that is, from 270° to 90° counterclockwise. The distributions of circumferential WSS at axial sections close to branch outlets follows cardioids-like pattern.

In order to get stable values, WSS is averaged peripherally for brachiocephalic left carotid, and left subclavian arteries. An area weighted average is obtained for 10 arc sections over each branch. The arch sections are essentially of arc length of 2.6 mm. The peripherally average WSS for brachiocephalic, left carotid and left subclavian arteries is presented in Figure 5(g). To avoid errors in averaging due to extreme WSS values close to branch entry and exit, WSS values close to the branch entry and exit regions are excluded from averaging. The values of mean WSS display limited variation along each branch which is about 3.5 Pa for brachiocephalic, 2.5 Pa for left carotid, and 1.5 Pa for left subclavian branches. As the lower WSS is blamed for the development of atherosclerotic plaques, the brachiocephalic artery displays more vulnerability to the development of plaques than the two others arteries. This conclusion is in agreement with earlier works [2, 3], where the brachiocephalic artery is determined as one of the predominant sites for localization of atherosclerosis plaque. However, Figure 5(g) shows a contradictory relation in a first glance, which indicates an average WSS can behave completely different than the local one. Also, an overall view through Figures 4(a)–4(d) and Figures 5(a)–5(g) indicates that the variation of WSS from distal to proximal side of arteries could be more relevant factor than WSS for the development of plaques. This variation is significantly large for the brachiocephalic artery. It should be noted that there are several other factors not studied here such as the geometry of aortic arch (patient-specific data associated with the shape of arch and branches with local details of the wall surface) and the flexibility of the aorta wall. Such factors will be studied through our next papers.

The mass flow rate across branch outlets followed distribution similar to outlet boundary condition. The flow rates were fairly the same in all three branches as it was observed for WSS, too. The reason behind similarity of mass flow rates is also the fact that the actual boundary condition was determined from equal mass flow rate condition.

### 3.2. Effect of Hypotension and Hypertension on the Distributions of Flow Field and WSS

**Figure 6:**
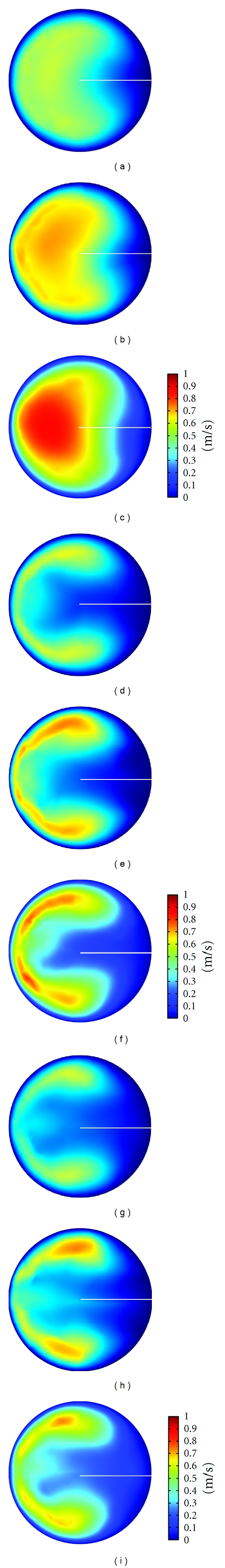
Axial velocity distributions at the axial cross-sections (a, b, c) A, (d, e, f) B, and (g, h, i) C (as shown in Figure 3(a)) through aortic arch at time *t* = 0.18 s, where a, d, and g correspond to hypotension; b, e and h correspond to normal pressure; c, f and i correspond to hypertension.

**Figure 7:**
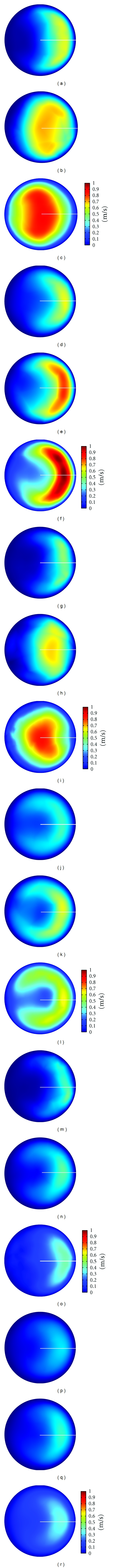
Axial velocity distributions at the axial cross-sections (a, b, c) D, (d, e, f) E, (g, h, i) F, (j, k, l) G, (m, n, o) H, (p, q, r) I (as shown in Figure 3(a)) through aortic arch at time *t* = 0.18 s, where a, d, g, j, m, and p correspond to hypotension; b, e, h, k, n, and q correspond to normal pressure; c, f, I, l, o and r correspond to hypertension.

The effect of pressure on flow parameters is studied by changing the dimensions of the aorta model based on the pressure-lumen area relation in aorta from Towfiq et al. [9]. Moreover, the boundary conditions are also modified to mimic hypotension and hypertension pressure regimes. The flow distributions in coronal plane are shown in Figures 2(b) and 2(d), while axial flow distributions across sections A–I (as shown in Figure 3(a)) are demonstrated in Figures 6 and 7. The distributions of pressure under the regimes of hypotension and hypertension were qualitatively identical to normal pressure case (shown in Figure 2(a)) and thus are not discussed here.

The flow distributions demonstrated in Figures 2(b), 2(c), and 2(d) are qualitatively identical. Like the velocity distribution across the coronal planes, the axial velocity distributions in respective axial sections shown in Figures 6 and 7 are also qualitatively identical. However, the comparison revealed that the velocity distributions are quantitative different and directly influenced by the hypotension and hypertension pressure regimes. The scale of velocity magnitude is high for hypertension case than that in hypotension and normal pressure cases. The velocity distributions in sagital and axial planes are most pronounced and amplified for hypertension case. The maximum velocity magnitude of 1.1 m/s observed in the hypertension case is 51% lower than the maximum velocity of hypotension case. Also the maximum velocity magnitude of 0.72 m/s observed in the normal pressure case is about 26% lower than the maximum velocity of hypotension case. Highly accelerated axial flow under hypertension regime is also evident in the axial flow distributions in aortic arch and three branches demonstrated in Figure 6 and Figure 7, respectively. The velocity distributions at sections A (Figures 6(a)–6(c)), D (Figures 7(a)–7(c)), and F (Figures 7(j)–7(l)) for hypertension case are almost parabolic with maximum velocity at the localized at the centre of the respective sections. On the other hand, the axial velocity distributions at sections A, D, and F for hypotension and normal case are nonuniform and with spatial nonuniformity. Higher axial velocity in normal pressure and hypertension case implicate dominant axial flow. Due to the dominant axial flow, the secondary flow patterns in hypotension are weaker while during hypertension secondary flow patterns intensify. The spatial variation in velocity fields are expected to affect the WSS distributions.

Within the branches, the flow is skewed toward the proximal walls, and the effect of hypotension and hypertension affects both the profile and the magnitude of velocity. Again the velocity profiles for hypertension case are more pronounced and amplified. Also amongst all the axial sections, maximum velocity magnitude is observed in section E (Figures 7(g)–6(i)), which lies in vicinity of outlet of brachiocephalic artery, for hypertension case. 

**Figure 8:**
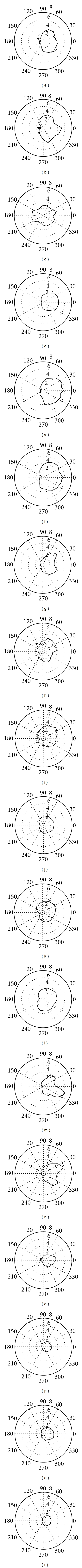
Polar plot of peripheral WSS distribution at the axial cross-sections (a, b, c) D, (d, e, f) E, (g, h, i) F, (j, k, l) G, and (m, n, o) H, and (p, q, r) I (as shown in Figure 3(a)) through aortic arch at time *t* = 0.18 s. Where a, d, g, j, m, and p correspond to hypotension; b, e, h, k, n, and q correspond to normal pressure; c, f, I, l, o, and r correspond to hypertension.

The effect of pressure regimes on WSS is closely investigated. The peripheral WSS for hypotension, normal pressure and hypertension is presented in form of polar plots in Figure 8. It can be noticed in Figure 8 that the magnitude of peripheral WSS does not change proportional to the pressure regimes. The WSS magnitude under hypertension condition is lower than that observed for normal cases while qualitatively the distributions are similar. Interestingly, for hypertension case, the values of WSS shown in Figure 8(c), 8(i), and 8(o) are, respectively, lower than WSS shown in Figure 8(b), 8(h), and 8(n). These correspond to the amplified velocity observed in Figure 7(c), 7(i), and 7(o). Relatively lower WSS at the branch entry regions and inner aortic arch may play a role in altering vascular biology and the localization of atherosclerosis [4, 6]. 

**Figure 9:**
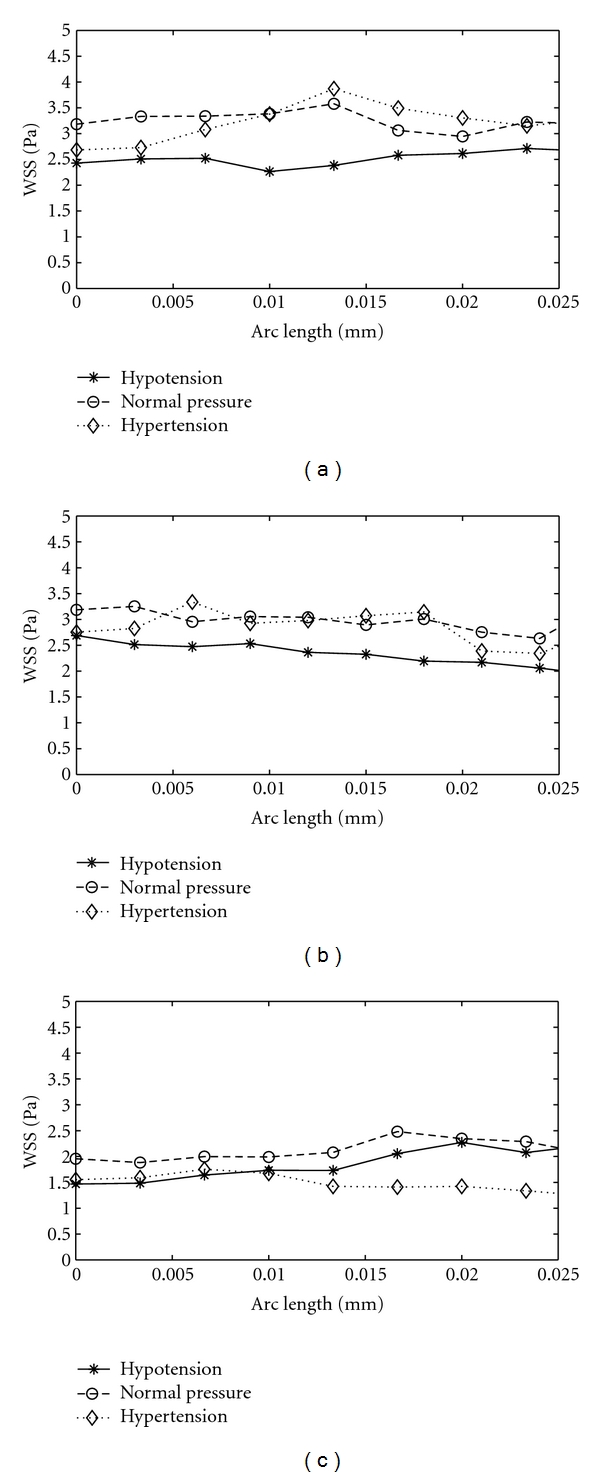
The average WSS along the (a) brachiocephalic, (b) left carotid, and (b) left subclavian branches of geometry G3 at peak systolic pressure of 80 mmHg (hypotension), 100 mmHg (normal), and 120 mmHg (hypertension) conditions.

The aorta is under greater peripheral stresses during hypertension. Dabagh et al. [29] have reported that due to the change in the morphology of endothelial cells higher rates of mitosis or apoptosis are expected, which increases the number of LDL pathways over endothelium. Thus an elevated risk of developing atherosclerotic plaques is anticipated under hypertensive conditions. It is also instructive to look at the variation of mean WSS through different sections along the branches. In order to get stable values, WSS is averaged along the length of the brachiocephalic, left carotid, and left subclavian branches for aorta model in Figure 9. It can be seen that the levels of WSS corresponding to hypertension do not considerably surpass those of normal pressure along each branch, but they may even go lower. As mentioned earlier, this can potentially boost the penetration of LDL macromolecules across the arterial wall. Figure 9 indicates that the average peripheral WSS is almost invariant along the branch though its peripheral distribution is not uniform. Note that the hypertensive wall is under higher strain while its shear stress is either unchanged or lower than what normally exist. According to Dabagh et al. [29], this can potentially boost the penetration of LDL macromolecules across the arterial wall.

## 4. Conclusions

In the present study, an idealized model of human aorta was reconstructed with measurements from the literature. Pulsatile blood flow in aorta model was simulated with FEM-based numerical solver. The aorta model along with boundary condition was modified to mimic the effect of hypotension and hypertension. The flow dynamic in the work displayed a reasonable qualitative agreement with other earlier works. Figures 4, 5, and 8 illustrate extremely low WSS at outer wall of aortic arch, proximal wall of branches, entry region of branches, and inner aortic arch. Low and oscillating shear stress are associated with risk of developing atherosclerosis, thus the listed sites are susceptible to localization of atherosclerosis. This conclusive observation is also consistent with earlier studies. The study has provided the fundamental understanding of the flow dynamics dependence on the artery geometry and the blood pressure in human aorta. Results reveal that the hemodynamics is significantly affected by the anatomy of aorta, which emphasizes on the patient-specific factor of the aorta geometry in developing atherosclerosis. The anatomy and orientation of branches have asignificant role in flow distribution and flow dynamics. On the other hand, the blood pressure affects the site of low WSS. It also appeared that the magnitude of WSS decreases under hypertension. It is associated with the deformation of aorta under high pressure. Our simulations were based on pressure-inlet boundary condition in the inlet and pressure-outlet boundary condition at the outlets, which reproduced developed flow profiles from the inlet section. The results show the fact that flow dynamics may be more complex when the geometry of aorta varies under pressure. A very important feature for our future study is the coupling the blood flow in aorta with wall deformation under elevated pressures to analyze the blood hemodynamics more precisely. To overcome limitations offered by idealized aorta models, computed-tomography- (CT-) scans-based realistic aorta will be used in future simulations.

## Figures and Tables

**Table 1:** Details of computational geometries measure in mm under normal pressure conditions.

Artery geometric measure (in mm)		Geometry
Ascending aorta	Lumen diameter (*B*_1_)	25.0
	Length (*L*_1_)	18.0
Aortic arch	Arch radius (*R*)	32.5
Descending aorta	Lumen diameter (*B*_2_)	25.0
	Length (*L*_2_)	75.0
	Lumen diameter (*B*_2_′)	20.0
Brachiocephalic artery (BA)	Diameter (*A*_1_)	8.8
	Length (*L*_1_)	28.0
	Diameter (*A*_1_′)	8.8
Left common carotid artery (LCA)	Diameter (*A*_2_)	8.8
	Length (*L*_2_)	28.0
	Diameter (*A*_2_′)	8.5
Left subclavian artery (LSA)	Diameter (*A*_3_)	9.9
	Length (*L*_3_)	28.0
	Diameter (*A*_3_′)	9.9
Distance between BA and LCA (*d*_1_)		3.6
Distance between LCA artery and LSA (*d*_2_)		4.7

**Table 2:** Values of coefficients for polynomial used as pressure waveform (×10^5^).

*C*_1_	*C*_2_	*C*_3_	*C*_4_	*C*_5_	*C*_6_	*C*_7_	*C*_8_
0.5601	0.1882	−1.4424	1.2239	−0.426	0.0664	−0.00492	0.000432

## References

[1] Caro CG, Fitz-Gerald JM, Schroter RC (1969). Arterial wall shear and distribution of early atheroma in man. *Nature*.

[2] Caro CG, Fitz-Gerald JM, Schroter RC (1971). Atheroma and arterial wall shear. Observation, correlation and proposal of a shear dependent mass transfer mechanism for atherogenesis. *Proceedings of the Royal Society of London Series B*.

[3] DeBakey ME, Lawrie GM, Glaeser DH (1985). Patterns of atherosclerosis and their surgical significance. *Annals of Surgery*.

[4] Ku DN, Giddens DP, Zarins CK, Glagov S (1985). Pulsatile flow and atherosclerosis in the human carotid bifurcation. Positive correlation between plaque location and low and oscillating shear stress. *Arteriosclerosis*.

[5] Nerem R, Bevan JA, Kaley G, Rubany GM (1995). Atherosclerosis and the role of wall shear stress. *Flow Dependent Regulation of Vascular Function*.

[6] Tarbell JM (2003). Mass transport in arteries and the localization of atherosclerosis. *Annual Review of Biomedical Engineering*.

[7] Zarins CK, Giddens DP, Bharadvaj BK, Sottiurai VS, Mabon RF, Glagov S (1983). Carotid bifurcation atherosclerosis. Quantitative correlation of plaque localization with flow velocity profiles and wall shear stress. *Circulation Research*.

[8] Fry DL (1968). Acute vascular endothelial changes associated with increased blood velocity gradients. *Circulation Research*.

[9] Towfiq BA, Weir J, Rawles JM (1986). Effect of age and blood pressure on aortic size and stroke distance. *British Heart Journal*.

[10] Utepov YY (1997). Correlation between anatomic parameters of the aorta and manifestations of atherosclerosis. *Bulletin of Experimental Biology and Medicine*.

[11] Kilner PJ, Yang GZ, Mohiaddin RH, Firmin DN, Longmore DB (1993). Helical and retrograde secondary flow patterns in the aortic arch studied by three-directional magnetic resonance velocity mapping. *Circulation*.

[12] Gao F, Guo Z, Sakamoto M, Matsuzawa T (2006). Fluid-structure interaction within a layered aortic arch model. *Journal of Biological Physics*.

[13] Kim T, Cheer AY, Dwyer HA (2004). A simulated dye method for flow visualization with a computational model for blood flow. *Journal of Biomechanics*.

[14] Mori D, Hayasaka T, Yamaguchi T (2002). Modeling of the human aortic arch with its major branches for computational fluid dynamics simulation of the blood flow. *JSME International Journal, Series C*.

[15] Morris L, Delassus P, Callanan A (2005). 3-D numerical simulation of blood flow through models of the human aorta. *Journal of Biomechanical Engineering*.

[16] Nakamura M, Wada S, Yamaguchi T (2006). Computational analysis of blood flow in an integrated model of the left ventricle and the aorta. *Journal of Biomechanical Engineering*.

[17] Park JY, Park CY, Hwang CM, Sun K, Goo Min B (2007). Pseudo-organ boundary conditions applied to a computational fluid dynamics model of the human aorta. *Computers in Biology and Medicine*.

[18] Shahcheranhi N, Dwyer HA, Cheer AY, Barakat AI, Rutaganira T (2002). Unsteady and three-dimensional simulation of blood flow in the human aortic arch. *Journal of Biomechanical Engineering*.

[19] Taylor CA, Hughes TJR, Zarins CK (1998). Finite element modeling of three-dimensional pulsatile flow in the abdominal aorta: relevance to atherosclerosis. *Annals of Biomedical Engineering*.

[20] Dabagh M, Jalali P, Konttinen YT, Sarkomaa P (2008). Distribution of shear stress over smooth muscle cells in deformable arterial wall. *Medical and Biological Engineering and Computing*.

[21] Khanafer KM, Gadhoke P, Berguer R, Bull JL (2006). Modeling pulsatile flow in aortic aneurysms: effect of non-Newtonian properties of blood. *Biorheology*.

[22] Conlon MJ, Russell DL, Mussivand T (2006). Development of a mathematical model of the human circulatory system. *Annals of Biomedical Engineering*.

[23] Li K-J (2004). *Dynamics of the Vascular System*.

[24] Ku DN (1997). Blood flow in arteries. *Annual Review of Fluid Mechanics*.

[25] Moayeri MS, Zendehbudi GR (2003). Effects of elastic property of the wall on flow characteristics through arterial stenoses. *Journal of Biomechanics*.

[26] Zeng D, Boutsianis E, Ammann M, Boomsma K, Wildermuth S, Poulikakos D (2008). A study on the compliance of a right coronary artery and its impact on wall shear stress. *Journal of Biomechanical Engineering*.

[27] Zhao SZ, Xu XY, Hughes AD (2000). Blood flow and vessel mechanics in a physiologically realistic model of a human carotid arterial bifurcation. *Journal of Biomechanics*.

[28] Wen CY, Yang AS, Tseng LY, Chai JW (2010). Investigation of pulsatile flowfield in healthy thoracic aorta models. *Annals of Biomedical Engineering*.

[29] Dabagh M, Jalali P, Tarbell JM (2009). The transport of LDL across the deformable arterial wall: the effect of endothelial cell turnover and intimal deformation under hypertension. *American Journal of Physiology*.

